# Anatomic Relationships of the Distal and Proximal Radioulnar Joints Articulating Surface Areas and of the Radius and Ulna Bone Volumes – Implications for Biomechanical Studies of the Distal and Proximal Radioulnar Joints and Forearm Bones

**DOI:** 10.3389/fbioe.2016.00061

**Published:** 2016-07-13

**Authors:** Paul S. C. Malone, Oliver G. Shaw, Vivien C. Lees

**Affiliations:** ^1^Department of Plastic Surgery, Institute of Inflammation and Repair, Wythenshawe Hospital, University Hospital of South Manchester NHS Foundation Trust, University of Manchester, Manchester, UK; ^2^Department of Plastic Surgery, Nottingham University Hospitals NHS Trust, Nottingham, UK

**Keywords:** biomechanics, anatomy, radius, ulna, PRUJ, DRUJ, joint surface areas, bone volumes

## Abstract

**Background:**

Previous work from this laboratory has evidenced the biomechanical role of forearm osseoligamentous structures in load transfer of applied forces. It has shown that forces transmitted across the distal radioulnar joint (DRUJ) and proximal radioulnar joint (PRUJ) are similar, though not identical, under axial loading conditions. The purpose of the study was to assess the articulating surface areas of the radioulnar joints and the volumes of the forearm bones addressing the hypothesis that there may be anatomic adaptations that reflect the biomechanical function of the integrated forearm unit.

**Methods:**

The articulating surface areas of PRUJ and DRUJ were assessed using a laser scanner in 24 cadaver forearms. The articulating joint surfaces were additionally delineated from standardized photographs assessed by three observers. The surface areas of matched pairs of joints were compared on the null hypothesis that these were the same within a given forearm specimen. An additional 44 pairs of matched forearm bone volumes were measured using water displacement technique and again compared through statistical analysis (paired sample *t*-test and Bland–Altman analysis).

**Results:**

The findings of this study are that the articulating surface areas of the DRUJ and PRUJ as well as the bone volumes are significantly different and, yet, strongly correlated. The paired sample *t*-test showed a significant difference between the surface areas of the DRUJ and PRUJ (*p* < 0.05). The PRUJ articulating surface area was marginally larger than the DRUJ with a PRUJ:DRUJ ratio of 1.02. Paired sample *t*-test showed a significant difference between the two bone volumes (*p* < 0.01) with a radius to ulna bone volume ratio of 0.81. When the olecranon was disregarded, radius volume was on average of 4% greater than ulna volume.

**Conclusion:**

This study demonstrates and defines the anatomical relationships between the two forearm bones and their articulating joints when matched for specimen. The data obtained are consistent with the theory of integrated forearm function generated from published biomechanical studies.

## Introduction

Throughout evolution, the forearm has developed into a highly complex and versatile part of the human anatomy. Elbow and wrist joints act in unison to facilitate placement of the hand in 3D space and prehension of objects from the environment. Improved independence of wrist and forearm rotation is thought to have occurred alongside brain development and facilitated primate brachiation (ability to swing through trees) and food gathering, while also permitting tool handling in the later stages of hominid evolution (Almquist, 1992). Understanding the anatomical structure and function of the forearm is important for those treating disorders of the forearm and especially for surgeons operating on the radius and/or ulna and their articulating joints. Forearm fractures and dislocations with ligamentous injury can affect the proximal radioulnar joint (PRUJ) and distal radioulnar joint (DRUJ) substantially, reducing forearm motion and producing pain (Crisco et al., 2007).

There is now a body of work in the scientific literature detailing the biomechanical properties of the functioning forearm unit with particular interest having focused on the DRUJ over the past two decades (Huang and Hanel, 2012). Originally, it was thought that the DRUJ simply facilitated forearm rotation, and it was common practice to remove the ulna head when that joint became pathologically compromised (Bowers, 1999). Subsequent cadaver-based studies have shown that force does pass across the DRUJ under specified loading conditions (Linscheid, 1992; Ischii et al., 1998). Force transmission profiles were detailed with respect to forearm pronosupination under a range of loading conditions (Shaaban et al., 2004). Changes in contact surface area within the DRUJ under these same conditions were detailed (Shaaban et al., 2007). This work also detailed the strains transmitted in the radius and ulna under the same loading conditions detailing a reciprocating system of load transfer that was compromised by removal of the ulna head (Shaaban et al., 2006). It was later shown that force would transmit through the PRUJ (Morrey et al., 1988). The coordinated movement (kinematics) of the DRUJ and PRUJ in 3D space was investigated (Baeyens et al., 2006). Work from our own laboratory (Malone et al., 2015) detailed the relationship of force transmission in simultaneous measurements on the DRUJ and PRUJ demonstrating almost identical force transmission profiles and contact areas profiles in the two joints and further detailed the force transmission characteristics of the component parts of the interosseous membrane linking the radius and ulna.

The impact of this body of work, detailing functional anatomy of the forearm and its biomechanical properties, has been to inform the development of operative procedures that preserve or aim to reconstruct the existing anatomy of the forearm unit leading to better clinical outcomes for patients. Part of this has been in the development of prosthetic arthroplasty for DRUJ replacement (Laurentin-Perez et al., 2008; Lees, 2015). Specific measurements were undertaken of ulna head mechanics in the development of one of the available unicomponent prostheses (Gordon et al., 2003, 2006).

It appears from the various studies undertaken on the kinematics and kinetics of the forearm joints and bones that there is a relationship in the magnitude of force that is transmitted under conditions of applied load and that simultaneous forces crossing the PRUJ and DRUJ are similar. Given their similar force transmission profiles it seemed possible – even logical – that there would be a relationship in the contact surface areas of the PRUJ and DRUJ and, potentially, similarities in the component radius and ulna bone volumes of the forearm.

Although radial head volume and articular surface area of the coronoid process have been measured (Guitton et al., 2010), there appears to be a lack of data concerning either the volumes of the radius and ulna or the surface area association between the PRUJ and DRUJ hemi-joints.

If the biomechanical model envisaging the forearm as having load-bearing functions is true then it could be that there are anatomic adaptations reflecting the reciprocating pattern of load transfer between the radius and ulna. This study was designed to measure the articulating surface areas of the PRUJ and DRUJ and the radius and ulna bone volumes to determine their relationship and the consistency of that relationship.

The purpose of this investigation was to see whether or not there are anatomic correlates of the biomechanical functions of the forearm. The importance of the information is that it would provide additional evidence for the theory of integrated forearm function and this, in turn, determines the direction of surgical design, implant, and prosthetic development.

## Materials and Methods

### Measurement of Articulating Surface Areas of the DRUJ and PRUJ

The articulating surface areas of the DRUJ and PRUJ were measured on 24 cadaveric specimens. For the DRUJ, this was the sigmoid notch of the radius, and for the PRUJ, this was the radial notch of the ulna. Fresh-frozen, cadaver upper extremities were obtained from the LifeLegacy Foundation, Arizona (www.LifeLegacy.org) and were handled according to local ethical guidelines (North Manchester Research Ethics Committee, UK). Cadaver limbs from deceased patients of 18–65 years age were included, and patients with the history of upper limb pathology were excluded. Pre-dissection radiographs excluded osteoarticular pathology. Specimens were stored at −40°C and gradually thawed according to a standard protocol.

Articulating surfaces of the specimens were prepared for 3D scanning by dissecting off all ligaments and soft tissues. This required a combination of sharp dissection and the use of a dissolution agent. A concentrated solution of biological detergent (Ariel™) was used to preserve clearly defined edges of the articulating surfaces of each joint. The specimens were soaked overnight and were then cleared of washing agent and loose soft tissue.

**Figure 1:**
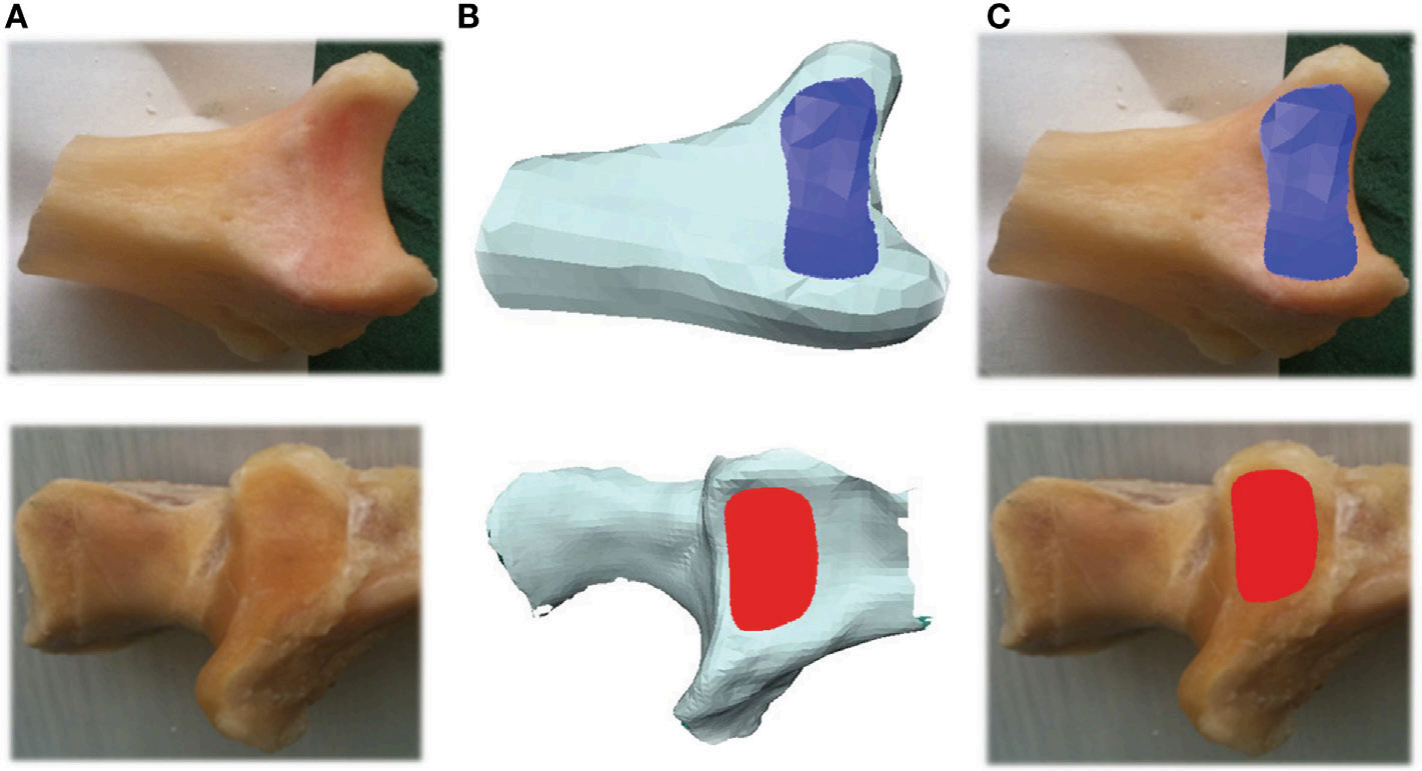
**Photograph of PRUJ and DRUJ being analyzed (A)**. 3D image of radioulnar joint as seen within Avizo^®^ program **(B)**. Articulating surface area of radioulnar joint superimposed onto photograph **(C)**. DRUJ (blue), distal radioulnar joint; PRUJ (red), proximal radioulnar joint.

Digital photographs of each joint were taken after disarticulation. The purpose of taking these images was to allow comparison with the laser scan image to ensure that the laser image accurately captured the visible joint surface so that laser imaging could, therefore, be regarded as a valid technique. The imaging involved capture of the articulating surface of the sigmoid notch of the distal radius (DRUJ) and the radial notch of the proximal ulna (PRUJ). This allowed reference images of each of the 24 paired PRUJ and DRUJ to be obtained (Figure 1A). The boundaries of each of the joints were independently outlined on the photographs by three assessors and then compared for interobserver variance. This process was repeated further two times over 3 weeks to reduce intraobserver variance. This was a qualitative process in which consistency in delineating the articulating surface of the joint was gained with the final version taken as the reference against which the scan was compared.

Three-dimensional laser images were obtained using a hand-held 3D scanning device: the Omega Scanner^®^ (Product No. OMG-00301), originally designed for the assessment of contour on amputee limbs. This was calibrated prior to use. Reflective surface markers were placed on the specimens allowing standardization of the surfaces being scanned to within an error rate of 0.5 mm^2^. Settings were selected for: “one-part scan; scan-type, knee; surface-type, medium; scan-length, 150 mm.”

Avizo 6.3.1^®^ software was used to visualize and analyze the 3D meshes of the PRUJ and DRUJ (.stl files; IT Services for Research, The University of Manchester, M13 9PL). The imported images were cropped to display the relevant anatomy being measured. For each joint scanned, the articulating surface areas were identified and traced within the Avizo^®^ program as a means of measuring the area. The program ParaView 3.98.1^®^ was used at the next stage to subdivide the trigonal mesh tiles produced by the Avizo^®^ program. While this does not alter the resolution of the original 3D images, it allows for much greater accuracy during the tracing and measurement process (Figure 1B). Having identified the boundaries of each of the proximal and distal joints, the areas were measured within Avizo^®^. At this point, these images were then superimposed onto their matching digital photograph by the way of further conformation of the boundaries of each joint (Figure 1C). (This was specifically done to ensure that each scan image properly represented the macroscopically visible joint edge on the photographed image. No laser image was manipulated as a consequence or adjustment made with all scanned images appropriately representing the visible joint surface).

### Relationship of Forearm Bone Volumes

Dry cadaver forearm bones were obtained from the University of Manchester Faculty Life Sciences’ Dissection Room and were accessed to measure and compare ulna and radius bone volumes. Specimens acquired had been stored as half skeletons and were examined for areas of damage and deformities prior to analysis. Damaged and porous bones were excluded from further analysis. Matched pairs of ulna and radius bones were identified using the university labeling systems. Correct matching of the pairs was further confirmed through manual articulation. Digital photographs of the 44 paired forearm bones were obtained for reference.

A fluid displacement system was used to measure the volume of each bone. The bone was fully submerged within a 500 ml measuring cylinder containing 450 ml of water. Only specimens that provided no ingress of water into the bony medulla *via* transosseous vascular foraminae were included in the study. The cylinder measuring scale was accurate to 0.5 ml. Following immersion of the bone, the rise in water level was recorded for each sample; with the gain representing the absolute bone volume. Reported data have been rounded up to the nearest whole millimeter reflecting the accuracy of the method. Each measurement was repeated, and the mean average was recorded. Radius and ulna volumes were analyzed, and the volumes for each specimen are plotted graphically with reference to the line of equality.

The same 44 paired forearm bones were remeasured a further time, with the ulna bone only being submerged to the base of the olecranon. This measured the volume of the ulna that solely contributes to the forearm unit.

### Statistical Methods

Pearson’s product–moment correlation coefficient was used to measure the strength and direction of association between joint surface areas and separately between bone volumes. Paired sample *t*-test was used to test the null hypothesis that there was no similarity in surface areas of the radial notch of the ulna and sigmoid notch of the radius and no similarity in radius and ulna bone volumes. Bland–Altman analysis was performed to further describe the difference between the groups. Data were analyzed using Statistical Product and Service Solutions (SPSS) software.

## Results

### Relationship of Articulating Surface Areas of the DRUJ and PRUJ

The articulating surface areas of the DRUJ (sigmoid notch of radius) and PRUJ (radial notch of ulna) were compared in 24 cadaver forearms. Data points were normally distributed and a paired sample *t*-test was applied.

**Figure 2:**
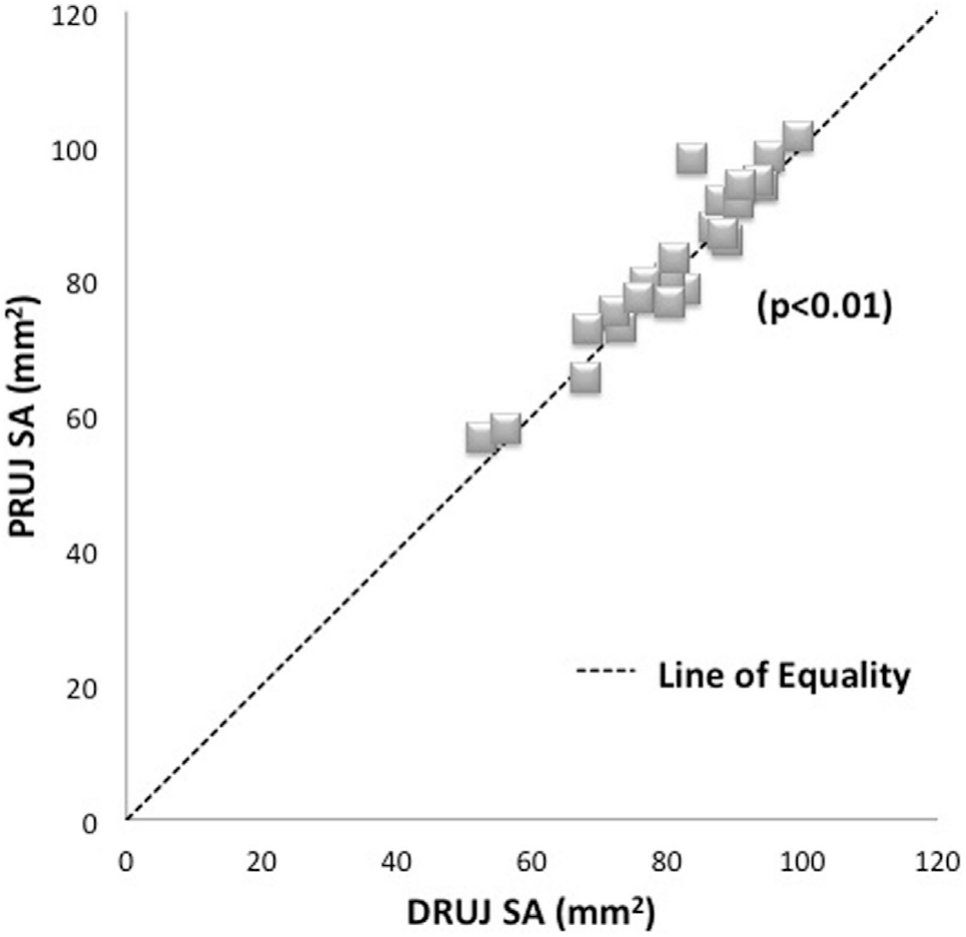
**Plot of matched specimens PRUJ and DRUJ surface areas demonstrating average PRUJ/DRUJ ratio of 1.02**. Each specimen pair is represented by one dot with several pairs showing as superimposed (diamonds). The line of equality (dashed line) represents the theoretical situation where articulating surface areas are exactly the same as one another. Proximal radioulnar joint = PRUJ; distal radioulnar joint = DRUJ.

The mean average PRUJ surface area was 82.56 mm^2^, which was marginally larger than the mean average DRUJ surface area of 80.75 mm^2^ (ranges 57.94–101.33 mm^2^ and 52.42–99.46 mm^2^, respectively) (Figure 2). Paired sample *t*-test showed a significant difference between the surface areas of the DRUJ and PRUJ (*p* < 0.05), with a PRUJ:DRUJ ratio of 1.02. Thus the radial notch surface area (PRUJ) was consistently and marginally larger than sigmoid notch surface area (DRUJ) (Figure 2).

Bland–Altman analysis. In this analysis, right and left arms from the same cadaver were treated as independent readings and showed a mean difference in surface area of 1.8 mm^2^ with 95% limits of agreement where the limits on agreement lay between −5.5 and 9.0. Hence, the difference in readings can be up to 9.0 mm^2^, which represents up to 11% of the joint surface area.

While the *t*-test showed that the values for the surface areas of the PRUJ and DRUJ are different, the average difference is only 2% with the PRUJ being on average marginally larger than the DRUJ. The maximum variation within the data for the group of arms is 11%, which would be expected, given the difference in body size between individuals. Taken together, these analyses show that the surface areas of the joint are reasonably similar in absolute values but significantly different in the sense that the PRUJ tends to be marginally, but consistently, albeit, marginally larger.

### Relationship of Forearm Bone Volumes

Analysis of the 44 matched pairs of forearm bones’ volumes showed that the radius volume was consistently less than ulna volume without exception.

**Figure 3:**
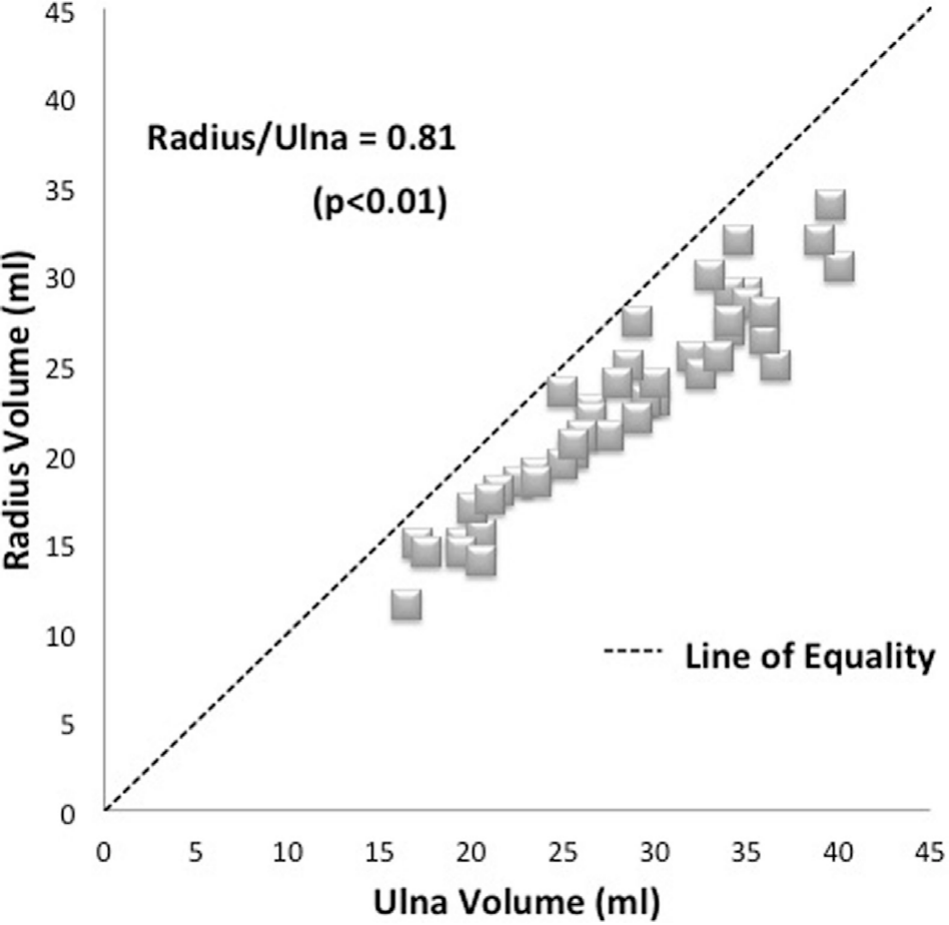
**Plot of matched specimens demonstrating average radius/ulna ratio of 0.81**. Each specimen pair is represented by one dot with several pairs showing as superimposed (diamonds). The line of equality (dashed line) represents the theoretical situation where bone volumes are exactly the same as one another.

Mean average ulna volume was 28 ml and mean average radius volume was 23 ml (ranges 16.5–40.0 ml and 14.0–34.0 ml, respectively). Paired sample *t*-test showed a significant difference between the two bone volumes (*p* < 0.01), with a radius: ulna ratio of 0.81. Thus, the radius volume was consistently smaller than the ulna volume (Figure 3).

Bland–Altman analysis showed a mean difference of 5.4 ml with 95% limits of agreement 1.3–9.5. The maximum variation between pairs of bones can be up to 9.5 ml, which represents up to 37% and reflects the difference in body size of the individual cadavers.

**Figure 4:**
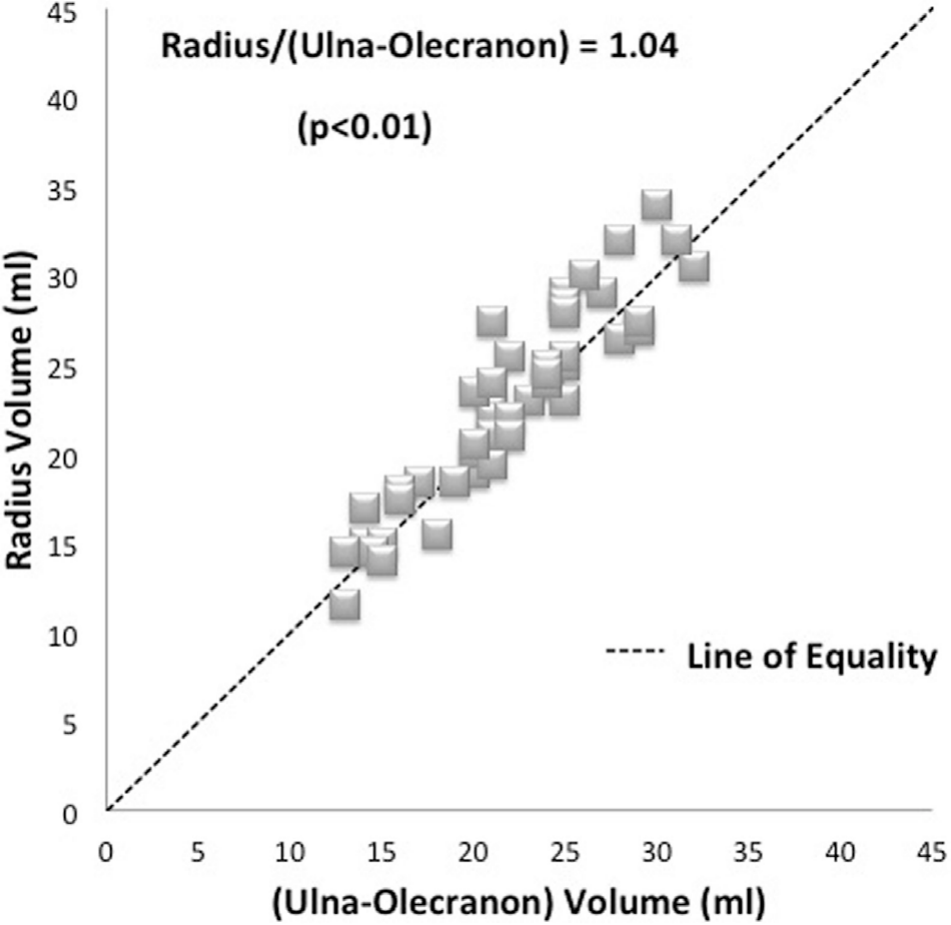
**Plot of radius and ulna bone volumes where the measured olecranon volume has been subtracted**. An average radius to forearm ulna component ratio of 1.04 is shown. Each specimen pair is represented by one dot with several pairs showing as superimposed (diamonds). The line of equality (dashed line) represents the theoretical situation where bone volumes are exactly the same as one another.

When the volume of the olecranon was subtracted, as modeled in the final part of the study, the total radius volume was found to be very similar to the remaining ulna volume. The correlation coefficient value (*r* = 0.93; *p* < 0.01). The mean radius volume was then demonstrated to be just 4% greater than the ulna (ratio 1.04 of radius compared with ulna volume) (Figure 4).

## Discussion

The findings of this study are that the articulating surface areas of the DRUJ and PRUJ as well as the bone volumes are significantly different and, yet, strongly correlated. The joint surface areas were measured at 82.56 mm^2^ for the radial notch of the ulna and 80.75 mm^2^ for the sigmoid notch of the radius. This represents a 2.2% difference in the surface areas, which is significantly different with the PRUJ being slightly larger than the DRUJ. The DRUJ figure is close to that reported in a previous study of Rozental et al. (2001), which showed the sigmoid notch articulation to be 79.78 mm^2^, although these authors did not measure the radial notch in their study. Their study analyzed the sigmoid notch in 20 cadaveric radius bones using CT images while our study has assessed 24 paired radioulnar joints using laser imaging. Reviewing previous work from our own laboratory, peak force transmission across the PRUJ has a slightly higher value than that of the DRUJ (Malone et al., 2015) representing 3.7% of the total. This makes the current observations with respect to the surface area measurements of interest within the context of our theory concerning integrated forearm function as there is a clear marginal difference of greater force transmission and surface area in the PRUJ compared to the DRUJ suggesting that the biomechanical measurements and the anatomical observations reported in this study may be inter-related.

The bone volume measurements demonstrated a clear and consistent relationship of volume of one bone to another within matched pairs, namely, that the radius was 81% of the volume of the ulna. This difference was statistically significant (*p* < 0.01). The correlation coefficient showed that this relationship was a consistent one (*r* = 0.95; *p* < 0.01). When the volume of the olecranon was subtracted, as modeled in the final part of the study, the total radius volume was found to be 4% greater than the remaining ulna volume with a correlation coefficient value (*r* = 0.93; *p* < 0.01). This lends some support to our hypothesis that the bones of the forearm excluding the elbow hinge are of similar volume because they transmit similar amounts of load.

The authors acknowledge certain limitations to their study. When measuring and analyzing both PRUJ and DRUJ using the Avizo^®^ computer software, several variables had to be minimized in order to produce reliable and non-biased findings. The edge of the proximal boundary of the sigmoid notch was difficult to delineate on some specimens. Collins and Vossoughi (2011) have demonstrated that the sigmoid notch is divided into two separate surfaces; an articulating and a non-articulating surface. The former is covered by cartilage that allows both rotational and gliding movements of the ulnar head within the DRUJ capsule. In this study, it was the articulating surface that was measured. By contrast, the borders of the radial notch of ulna were readily discerned due to the defined surrounding ligamentous attachments.

The Omega Scanner^®^ was designed for imaging limb amputations in preparation for prosthetic fitment. The technology has been used here to assess small (<10 cm^2^), concave, bony, and cartilaginous surfaces. Refractive interference from laser beam reflection of shinier surfaces of the joints was an initial problem overcome by dusting powder onto those surfaces. The scanner measurements were accurate to within 0.5mm^2^, which caused minor angulations of the images; this can be appreciated from Figure 1. The available software was for the assessment of surface area and contour but was specifically not designed for volume measurement. For this reason, bone volume was not assessed by the laser scanner in these studies. With respect to the bone volume measurements using water displacement methods, these measurements were judged accurate to within 0.5 ml (mean bone volume 25 ml, therefore, error of measurement was up to 2%).

In conclusion, the findings of this study further enhance and substantiate our understanding of the functional anatomy of the forearm unit. The data from this study are consistent with the hypothesis that there are anatomic adaptations of the radius and ulna specifically with respect to the articulating joint surface areas of the PRUJ (radial notch of ulna) and DRUJ (sigmoid notch of radius) that reflect their known function of facilitating pronosupination and load transfer. Biomechanical studies have similar, though not identical, force transmission profiles for these two joints (Shaaban et al., 2004, 2006; Malone et al., 2015), and it is instructive to find that the articulations have similar, though not identical, surface areas. When that part of the ulna, which contributes to the hinge of the elbow joint, is excluded, then the volumes of the radius and ulna that comprise the integrated forearm unit are similar from a clinical standpoint. This observation is consistent with the previous description of a reciprocating load transfer system between the radius and ulna (Shaaban et al., 2004). These findings are important as they suggest that previous observations and mechanical modeling by both ourselves and others are real and relevant. The data reported here will also be of assistance to those undertaking computer modeling of the forearm and should have practical impacts for those involved in prosthetic design.

## Human and Animal Rights

Ethical permission was granted by the UK National Research Ethics Committee (reference number 07/Q1406/7). This article does not contain any studies with live human or animal subjects.

## Author Contributions

PM – lead Researcher: project design, initiation, development, and running. OS – second researcher undertaking significant part of methods. VL – concept, supervision, preparation and editing of manuscript. All three authors had significant involvement with the writing and rewriting of article drafts, approve the submitted version, and agree to be accountable for all elements of the submitted work.

## Conflict of Interest Statement

The authors declare that the research was conducted in the absence of any commercial or financial relationships that could be construed as a potential conflict of interest.
