# Supplementary material for: Anatomic Relationships of the Distal and Proximal Radioulnar Joints Articulating Surface Areas and of the Radius and Ulna Bone Volumes – Implications for Biomechanical Studies of the Distal and Proximal Radioulnar Joints and Forearm Bones
Source: Front Bioeng Biotechnol. 2016 Jul 13;4:61. doi: 10.3389/fbioe.2016.00061 (PMC4942467; doi:10.3389/fbioe.2016.00061)
Supplement: Supplementary file 1 [file table_1.pdf]

Appendix – A table to show the joint surface area measurements and compared DRUJ:PRUJ ratios.

Specimen Name	Left DRUJ	Left PRUJ	% diff	DRUJ:PRUJ	Right DRUJ	Right PRUJ	% diff	DRUJ:PRUJ
01002	93.95	94.41	0.5	1.0	88.77	86.17	-3.0	1.0
02054	86.98	87.97	1.1	1.0	88.30	87.08	-1.4	1.0
10031	82.68	78.69	-5.1	1.0	80.20	76.73	-4.5	1.0
12008	80.18	80.31	0.2	1.0	80.96	83.43	3.0	1.0
12017	72.43	75.54	4.1	1.0	72.30	75.62	4.4	1.0
10033	52.42	56.66	7.5	1.1	56.16	57.94	3.1	1.0
09053	87.97	92.07	4.5	1.0	90.80	94.16	3.6	1.0
09072	94.95	98.63	3.7	1.0	99.46	101.33	1.8	1.0
102643	73.06	73.18	0.2	1.0	75.68	77.55	2.4	1.0
09074					67.79	65.50	-3.5	1.0
09075L	93.60	95.05	1.5	1.0				
010911L	76.79	79.75	3.7	1.0				
012749L	90.63	91.72	1.2	1.0				
071022R1					68.36	72.90	6.2	1.1
071022R2					83.58	98.26	14.9	1.2

DRUJ = Distal radioulnar joint; PRUJ = proximal radioulnar joint; SD= standard deviation;

% diff = percentage difference between ipsilateral PRUJ and DRUJ.

Mean average DRUJ:PRUJ ratio of 1:1.02 (range = 0.95-1.18)

Mean average DRUJ measurement = 80.75mm2 (SD = 11.95mm2)

Mean average PRUJ measurement = 82.56 mm2 (SD = 12.25mm2)
